# Supplementary material for: A Study of Opiate, Opiate Metabolites and Antihistamines in Urine after Consumption of Cold Syrups by LC-MS/MS
Source: Molecules. 2020 Feb 21;25(4):972. doi: 10.3390/molecules25040972 (PMC7070706; doi:10.3390/molecules25040972)
Supplement: Supplementary file 1 [file molecules-25-00972-s001.pdf]

Supplementary Materials

Article

A Study of Opiate, Opiate Metabolites and Antihistamines in Urine after Consumption of Cold Syrups by LC-MS/MS

Yao-Te Yen *, Yin-Jue Chang, Pin-Jung Lai, Chi-Lun Chang, Ting-Yueh Chen and San-Chong Chyueh

Department of Forensic Science, Investigation Bureau, Ministry of Justice, Xindian District, New Taipei City 23149, Taiwan; b98605015@ntu.edu.tw (Y.-J.C.); r02625039@ntu.edu.tw (P.-J.L.); b98605019@ntu.edu.tw (C.-L.C.); r96524028@ntu.edu.tw (T.-Y.C.)

* Correspondence: d05223113@ntu.edu.tw; Tel.: 011-886-2-29112241-3714

Table 1. MRM transitions, retention times, and tuning parameters of the analytes.

Compound	Precursor ion (m/z)	Product ion (m/z)	Retention time (min)	Frag-Mentor (V)	Collision Energy (eV)	Ionization mode
Morphine	286.2	165.0	3.7	139	48	Positive
		153.0			48	
6-Acetylmorphine	328.2	165.0	8.9	165	44	Positive
		211.0			28	
Morphine-3-β-D-glucuronide	462.2	286.2	2.8	175	32	Positive
		201.0			46	
Morphine-6-β-D-glucuronide	462.2	286.2	3.8	160	32	Positive
		190.9			56	
Codeine	300.2	165.0	7.8	140	44	Positive
		153.0			54	
Codeine-6-β-D-glucuronide	476.2	300.1	7.7	195	32	Positive
		225.1			40	
Chlorpheniramine	275.1	230.0	9.9	104	16	Positive
		167.0			48	
Carbinoxamine	291.1	167.0	10.0	104	36	Positive
		202.0			12	
Morphine-d_6	292.2	153.1	3.6	155	40	Positive
		181.0			52	
Codeine- d_6	306.2	218.1	7.7	165	28	Positive
		165.0			48	
Chlorpheniramine-d_6	281.2	230.0	9.9	99	12	Positive
		167.0			48	

Table 2. Slopes and MEs of urine matrix calibration curves using "dilute-and-shoot" analyses.

Matrix	Morphine	6-AM	M3G	M6G	Codeine	C6G	Chlorpheniramine	Carbinoxamine
Neat	0.026	0.039	0.019	0.138	0.012	0.056	0.017	0.019
1	0.027 (3.8) [a]	0.039 (0)	0.024 (26.3)	0.150 (8.7)	0.012 (0)	0.056 (0)	0.019 (11.8)	0.019 (0)
2	0.027 (3.8)	0.039 (0)	0.025 (31.6)	0.148 (7.2)	0.012 (0)	0.056 (0)	0.020 (17.6)	0.020 (5.3)
3	0.026 (0)	0.039 (0)	0.025 (31.6)	0.145 (5.1)	0.012 (0)	0.056 (0)	0.020 (17.6)	0.020 (5.3)
4	0.026 (0)	0.039 (0)	0.025 (31.6)	0.147 (6.5)	0.012 (0)	0.056 (0)	0.020 (17.6)	0.020 (5.3)
5	0.026 (0)	0.039 (0)	0.024 (26.3)	0.145 (5.1)	0.012 (0)	0.056 (0)	0.019 (11.8)	0.019 (0)
Mean value [b]	0.026	0.039	0.025	0.147	0.012	0.056	0.020	0.020
Precision [c] (%)	2.1	-	2.2	1.4	-	-	2.8	2.8

[a] ME shown in parentheses. [b] Mean value is the average of slope values of calibration curves in urine matrix 1 to 5. [c] Precision is the coefficient of variation of slope values of calibration curves in urine matrix 1 to 5.

Table 3. Accuracy (%) and precision (CV %) for QC in dilution integrity test. (n=3).

Compound	Morphine	6-AM	M3G	M6G	Codeine	C6G	Chlorphenir-amine	Carbinox-amine
**Accuracy for QC**								
**QC-Low**								
**1/20x**	94.0	103.5	96.3	96.6	103.9	102.0	105.9	103.9
**1/10x**	99.8	102.1	105.2	105.2	104.7	106.0	103.7	104.2
**1/2x**	105.7	105.1	105.1	106.3	104.9	102.9	105.9	106.2
**QC-Medium**								
**1/20x**	105.5	106.1	104.8	105.2	104.7	104.9	106.9	106.3
**1/10x**	94.7	101.4	101.0	97.0	106.2	99.0	103.5	106.0
**1/2x**	95.2	101.4	101.1	97.8	105.7	96.7	102.1	102.4
**QC-High**								
**1/20x**	96.4	104.4	105.5	103.1	104.6	103.6	105.1	106.0
**1/10x**	100.9	104.1	105.1	98.2	104.6	104.7	105.3	106.7
**1/2x**	95.3	104.8	102.8	100.3	103.4	104.7	104.8	103.9
**Precision for QC**								
**QC-Low**								
**1/20x**	4.0	1.8	3.8	3.8	1.6	0.7	1.9	1.7
**1/10x**	2.3	0.2	0.6	3.2	0.7	2.5	0.9	2.2
**1/2x**	2.6	3.1	1.8	1.6	1.2	2.8	2.4	3.8
**QC-Medium**								
**1/20x**	2.8	3.7	2.0	2.9	2.1	1.1	2.4	2.5
**1/10x**	1.4	1.2	0.5	4.8	3.2	2.6	1.1	1.1
**1/2x**	2.9	2.1	1.2	1.9	2.4	3.8	2.5	0.3
**QC-High**								
**1/20x**	5.2	2.8	3.0	1.7	1.9	2.0	2.8	1.5
**1/10x**	1.1	1.7	1.2	3.5	2.5	3.1	2.1	2.5
**1/2x**	3.3	2.2	2.7	2.8	2.4	2.6	1.9	3.5

Table 4. Stability assessment.

Compound	Day				Stability assessment	
	7	14	21	28	Mean value	Precision (%)
Morphine	20.4 ± 0.5	20.3 ± 0.8	20.7 ± 0.2	20.4 ± 0.5	20.5 ± 0.2	0.8
6-AM	19.6 ± 0.3	20.7 ± 0.2	19.3 ± 0.2	19.3 ± 0.4	19.7 ± 0.7	3.4
M3G	19.6 ± 0.6	20.6 ± 0.3	20.5 ± 0.4	20.7 ± 0.7	20.4 ± 0.5	2.5
M6G	19.6 ± 0.2	19.1 ± 0.1	20.6 ± 0.3	21.6 ± 0.7	20.2 ± 1.1	5.5
Codeine	20.1 ± 0.6	20.4 ± 0.2	20.0 ± 0.4	19.9 ± 0.8	20.1 ± 0.2	1.1
C6G	19.5 ± 0.0	19.2 ± 0.2	20.8 ± 0.1	20.5 ± 0.1	20.0 ± 0.8	3.9
Chlorpheniramine	20.3 ± 0.2	20.5 ± 0.4	20.4 ± 0.2	20.6 ± 0.4	20.5 ± 0.1	0.6
Carbinoxamine	19.7 ± 0.3	19.5 ± 0.1	19.3 ± 0.2	19.8 ± 0.1	19.6 ± 0.2	1.1

Note: Unit: ng/mL

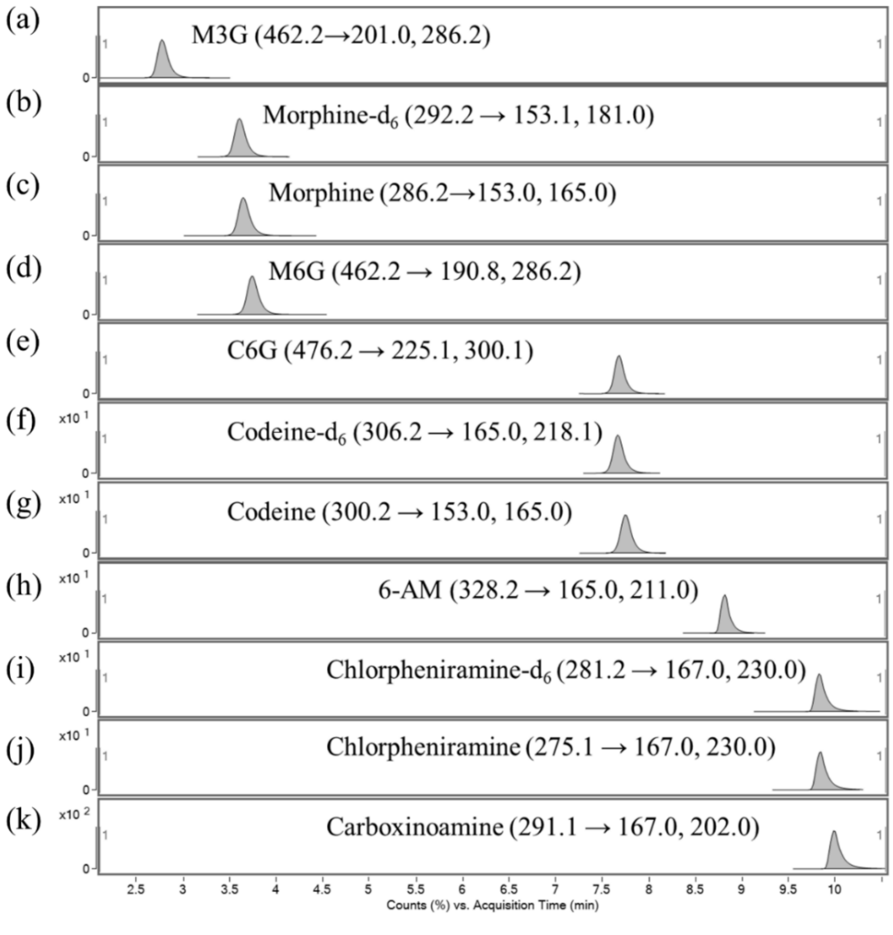

Figure 1. Chromatograms of analytes (1000 ng mL^{-1}) obtained using LC-MS/MS in extracted-ion mode.

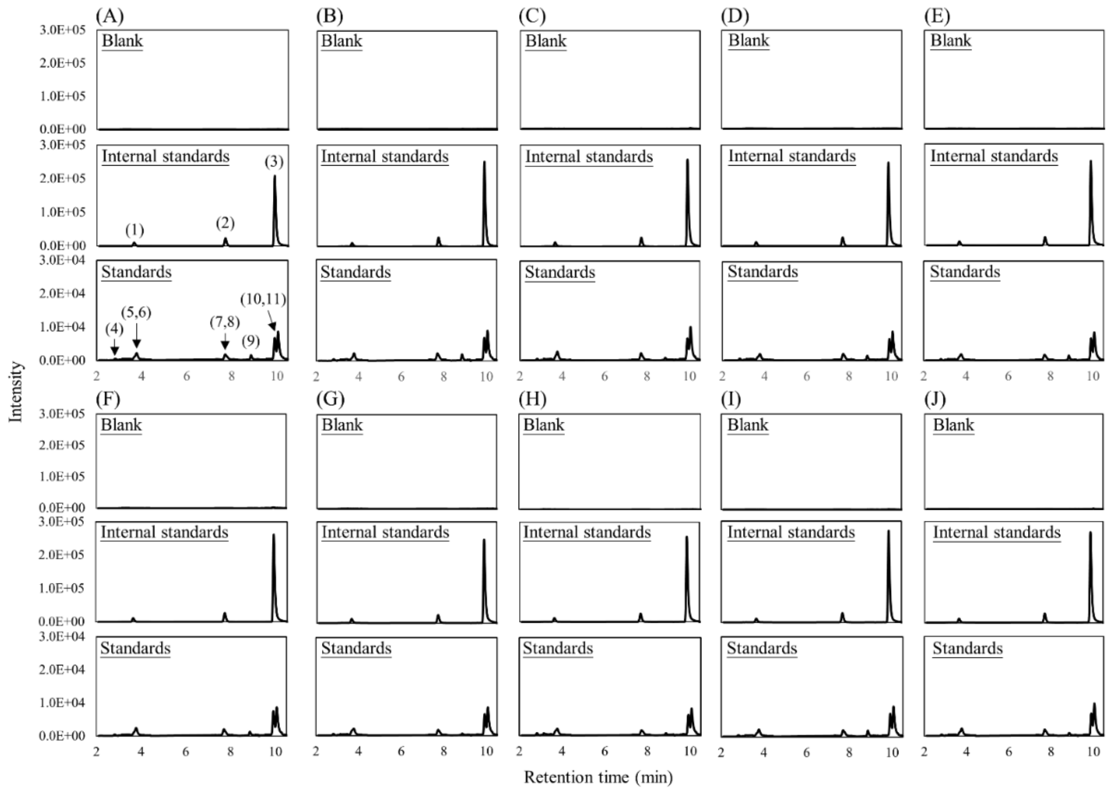

Figure 2. Chromatograms of ten lots of blank urine (A-F) and samples spiked with IS or LOQ-analytes in total-ion mode. (1) morphine-d6; (2) codeine-d6; (3) chlorpheniramine-d6; (4) M3G; (5) morphine; (6) M6G; (7) C6G; (8) codeine; (9) 6-AM; (10) chlorpheniramine; (11) carbinoxamine.
